# Lectures on the Theory and Practice of Physic

**Published:** 1845-01

**Authors:** 


### BIBLIOGRAPHICAL NOTICES.

*Lectures on the Theory and Practice of Physic.* By John Bell, M. D., Fellow of the College of Physicians of Philadelphia: Corresponding Secretary of the Philadelphia Medical College: Member of the American Philosophical Society, and of the Georgofili Society of Florence, etc. etc. And by William Stokes, M. D., Lecturer at the Medical School, Park Street, Dublin; Physician to the Meath Hospital, etc. etc. *Third Edition, enlarged and improved. In two volumes. 8vo.* Philadelphia. Ed. Barrington & George D. Haswell. 1845.

These are two ponderous volumes, making in all more than fifteen hundred pages, containing a vast amount of information on many of the most important subjects in medical science. The

*original* portion of this work, if we may be allowed the phrase, has undergone sundry transformations, and strange as it may seem, it has never yet been published in a distinct form in England: yet the name of Stokes has long been most popular there as elsewhere, although his lectures are strongly tinctured with the exploded doctrines of Broussais. We do not know, indeed, whether this did not obtain for them more ready currency in this country; for, certainly, nowhere were these doctrines—seductive in their simplicity, but as unfounded, as a whole, as they were seductive—so ardently and recklessly embraced, and so suddenly and unscrupulously abandoned. It was in the Medical Library of Dr. Dunglison that these lectures of Dr. Stokes were first collected and printed from the pages of a London medical periodical. Subsequently, they were reprinted, with other lectures by Dr. Bell, under the title of "*Stokes* and Bell's lectures;"—we mean the title placed upon the cover. In no great lapse of time the edition was exhausted; and then appeared two goodly sized volumes—and now we have what is termed a *third edition*, as it would be, indeed, were the title of the work retained with the precedence given to the distinguished European whose lucubrations constitute its basis; but in place of this, the gilt letters on the back now inform us that the work is "Bell & Stokes' Practice," (of medicine, of course:) instead, therefore, of its being the *third* edition of "Stokes & Bell," it is, in fact, the *first* of "Bell & Stokes."

We confess we do not exactly see either the object or the propriety of this change. We presume it has been made in consequence of the labours of the American Editor having attained such dimensions as to exceed those of the original author: and we know, that the example of Dr. Elliotson might be cited to vindicate even greater changes; for he, in his last edition of Blumenbach, boldly abandoned even the title of Blumenbach's physiology, and gave to it that of Elliotson's. Nor is this the first instance in which Elliotson has been taken as a prototype. But we think the practice is "more honoured in the breach than the observance."

Dr. Bell has bestowed much industry on the present edition. He has supplied many marked deficiencies in the former: for instance, we have now "*Diseases of the Organs of Generation*, in both sexes; several diseases of the *Respiratory Apparatus*, omitted before; *Tubercular Meningitis, Epidemic Meningitis; Scrofula; Syphilis;* the *Exanthemata*, greatly enlarged; *Rheumatism*, and *Gout*. The new matter added in these ways is fully equal, it is believed, to three hundred pages."

It is impracticable for us to give more than a general view of the nature of those additions. In the main they are judicious, and in accordance with the opinions of the day, and are highly creditable to Dr. Bell as a well read physician and able writer. We cannot avoid thinking, however, that he has displayed some partiality in his citation of authorities. It is difficult, perhaps, to avoid preferences. Favours received, or patronage bestowed; long continued personal friendship; early prejudice, &c., may warp the judgment and occasion misplaced laudation or neglect, despite the intentions of the purest minds. We could point out more than one extract, the selection of which has been suggested, we think, by indulgent partiality, and which appear to us so trite and common place, that we are surprised that any kindness of feeling should have prompted him to raise them to such prominent positions.

Dr. Bell has written extensively on various points of medical science and practice, and we do not marvel, therefore, to find frequent reference to his own productions; and although some may think these references are too numerous, or even deny to an author the privilege of referring to his own writings at all, for information which can be obtained in any of the standard works on the same subject, we can conceive of cases in which it may be necessary, in order to avoid extracting whole passages, or incurring the risk of being misunderstood.

Although Dr. Bell's as well as Dr. Stokes' part of the present publication is in the form of Lectures, the former we believe were never delivered, and are written in that style we presume to harmonize with the latter: perhaps, too, greater scope is afforded in that way for illustration. We cannot but regret, however, the evident haste to bring out these Lectures, which has precluded a careful correction of the press. In a work designed for extensive circulation, such expressions as these ought not to occur: "Kop and Otto, both speak in terms of the highest praise of the use *by* the conium maculatum, in scrofulous ophthalmia." "Another symptom very common in this disease is the great sensibility *of* the return to light." "Periodical fevers are most frequent and violent in climates and localities, *and* during or immediately following the season of the greatest contrasts, and alterations in the sensible states of the atmosphere." These, of course, will all be corrected in a future edition.

To conclude. Notwithstanding the errors to which we have referred, and some others that we might notice—mostly, however, of minor importance, the lectures of "Bell & Stokes" must be regarded as constituting an exceedingly valuable treatise on the Practice of Medicine. Those by our friend and fellow townsman, to which we more particularly refer, exhibit much learning and research, judicious discrimination, and a thorough acquaintance with the diseases and practice of this country. More need not be said to entitle him to the thanks of the profession for his share in this publication.

